# Epigenetic Regulation of a Disintegrin and Metalloproteinase (ADAM) Transcription in Colorectal Cancer Cells: Involvement of β-Catenin, BRG1, and KDM4

**DOI:** 10.3389/fcell.2020.581692

**Published:** 2020-09-11

**Authors:** Lina Sun, Baoyu Chen, Jiahao Wu, Chao Jiang, Zhiwen Fan, Yifei Feng, Yong Xu

**Affiliations:** ^1^Department of Pathophysiology, School of Biological and Basic Medical Sciences, Soochow University, Soochow, China; ^2^Institute of Biomedical Research, Liaocheng University, Liaocheng, China; ^3^Key Laboratory of Targeted Invention of Cardiovascular Disease and Collaborative Innovation Center for Cardiovascular Translational Medicine, Department of Pathophysiology, Nanjing Medical University, Nanjing, China; ^4^Department of Surgical Oncology, Affiliated Hospital of Nanjing University of Chinese Medicine, Jiangsu Province Hospital of Chinese Medicine, Jiangsu, China; ^5^The First School of Clinical Medicine, Nanjing Medical University, Nanjing, China; ^6^Department of General Surgery, The First Affiliated Hospital of Nanjing Medical University, Nanjing, China

**Keywords:** transcriptional regulation, colorectal cancer, matrix metalloproteinase, Wnt signaling, β-catenin, BRG1, KDM4, epigenetics

## Abstract

A disintegrin and metalloproteinase (ADAM) family of proteins play versatile roles in cancer development and progression. In the present study, we investigated the role of ADAM proteins in colorectal cancer (CRC) cell migration and invasion focusing on the epigenetic mechanism whereby ADAM transcription is regulated. We report that higher levels of ADAM10, ADAM17, and ADAM19 were detected in SW480 cells than in HCT116 cells. Expression levels of the same set of ADAMs were higher in human CRC biopsy specimens of advanced stages than in those of a less aggressive phenotype. Overexpression of ADAM10/17/19 in HCT116 cells enhanced, whereas depletion of ADAM10/17/19 in SW480 cells weakened, migration and invasion. ADAM expression was activated by the Wnt signaling pathway, which could be attributed to direct binding of β-catenin on the ADAM promoters. Mechanistically, β-catenin recruited the chromatin remodeling protein BRG1, which in turn enlisted histone demethylase KDM4 to alter the chromatin structure, thereby leading to ADAM transactivation. In conclusion, our data suggest that the Wnt signaling may promote CRC metastasis, at least in part, by recruiting an epigenetic complex to activate ADAM transcription.

## Introduction

Colorectal cancer (CRC) represents one of the deadliest forms of cancers worldwide with approximately 2 million projected new diagnoses each year (Bray et al., 2018). Implementation of early screening, advancement of surgical techniques, and personalized chemotherapeutic medications have considerably alleviated the mortality and morbidity of CRC patients. These improvements in CRC prevention and intervention notwithstanding, disease-free survival rate for patients diagnosed with advanced stages of CRC remains dreadfully low, with more than 800,000 CRC patients succumbing to this malicious disease in 2018 alone (Araghi et al., 2019). Malignant types of CRC are typically characterized by aggressive migration and invasion; by the time of diagnosis, a large fraction of the cancer cells have already metastasized to distal sites rendering surgical resection and chemotherapy less effective and dampening the long-term prognosis (Riihimaki et al., 2016). Clearly, in order to devise a viable interventional approach to treat CRC, it is essential to close the gap in our understanding of the molecular mechanism that governs the malignant migration and invasion of CRC cells.

A complicated network of signaling pathways, of which the Wnt signaling pathway is the best characterized, orchestrates the pathogenesis of CRC (Behrens, 2005). In normal intestinal epithelial cells, the Wnt signaling is turned off because its downstream mediator β-catenin becomes constitutively phosphorylated and consequently degraded by a so-called “destruction complex” consisting of two protein kinases (GSK3 and CK1), the adenomatous polyposis coli (APC) protein, the E3 ligase TrCP, and the scaffolding protein Axin2 (Stamos and Weis, 2013). Aberrant activation of the Wnt signaling (by APC loss-of-function mutations) leads to stabilization and subsequent nuclear translocation of β-catenin and boosts malignant transformation of intestinal epithelial cells. β-catenin mediates Wnt signaling to promote CRC development and progression primarily by dictating a proproliferative and prometastatic transcriptional program. For instance, β-catenin can directly activate the transcription of E-box repressors Snail, Slug, and Twist to promote epithelial–mesenchymal transition and facilitate metastasis (Yang et al., 2013). In addition, β-catenin enhances the mobility of metastasizing cancer cells by activating the transcription of a panel of different metalloproteinases (MMPs) to contribute to the remodeling of the extracellular matrix (Lowy et al., 2006; Kamino et al., 2011; Yang F. et al., 2019). Regulation of MMPs by several mechanisms including transcriptional activation via promoter and via miRNAs plays important roles in the remodeling of extracellular matrix (Surgucheva et al., 2003; Surgucheva et al., 2010).

A disintegrin and metalloproteinases (ADAMs) belong to a family of transmembrane proteolytic enzymes that play keys roles in cell proliferation, adhesion, migration, and invasion. ADAMs exert their wide-ranging effects by separating membrane-anchored proteins from their extracellular domains in a process termed “ectodomain shedding.” Previous investigations have implicated ADAMs in the development and progression of CRC (Przemyslaw et al., 2013). Park and Kim (2017) have reported that TLR4-initiated signaling activates the expression of ADAM10 and ADAM17 to promote glycolysis and lactate production fueling the malignant proliferation of CRC cells. Mustafi et al. (2017) have demonstrated that ADAM17 contributes to colorectal carcinogenesis by cleaving and thus releasing the pro-EGFR ligands. Histological examination of CRC biopsy specimens and profiling of blood samples collected from CRC patients both indicate that ADAM expression levels are altered during CRC pathogenesis (Duffy et al., 2009). It remains underexplored, however, how the expression levels of different ADAM proteins are modulated at the transcriptional level. In the present study, we investigated the epigenetic mechanism whereby ADAM expression is regulated in CRC cells. We report that ADAM10, ADAM17, and ADAM19 are direct transcriptional targets of β-catenin. β-catenin forms a transcription complex with the chromatin remodeling protein BRG1 and histone demethylase KDM4 to cooperatively activate ADAM transcription.

## Results

### Differential ADAM Expression Correlates With CRC Malignancy

**FIGURE 1:**
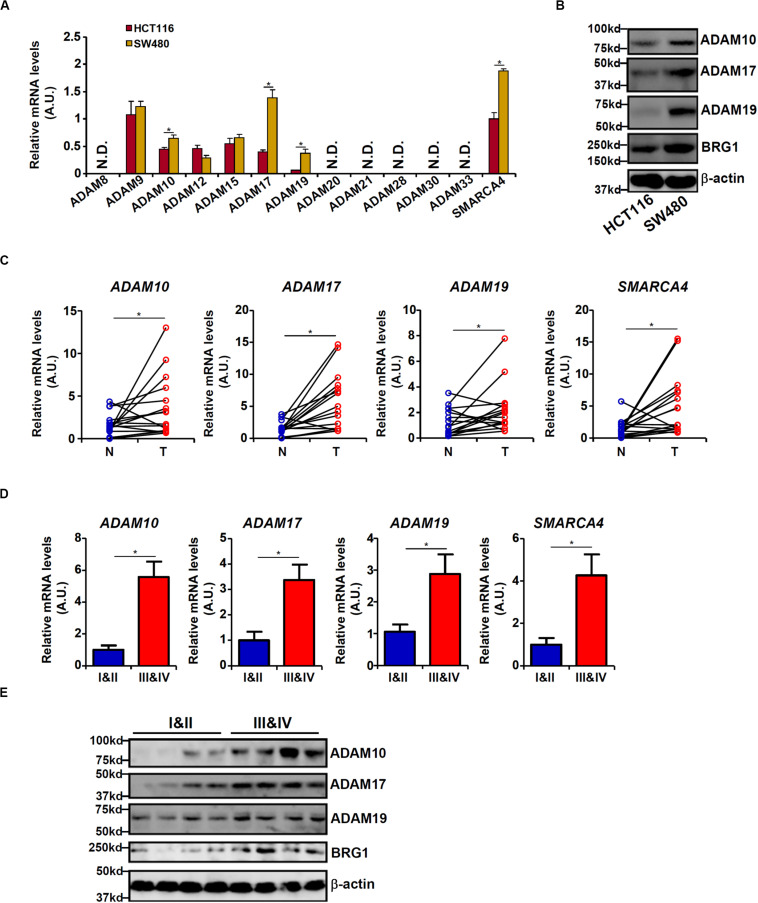
Differential ADAM expression correlates with colorectal cancer (CRC) cell malignancy. **(A,B)** Expression levels of ADAMs in SW480 cells and HCT116 cells were examined by qPCR and Western blot. **(C)** ADAM expression levels in human CRC biopsy (T) and paired non-tumorous (N) specimens were examined by qPCR. *N* = 14 cases. **(D,E)** ADAM expression levels in human CRC biopsy specimens of different grades were examined by qPCR and Western. *N* = 5 cases for each group. Data represent averages of three independent experiments, and error bars represent SEM (*p* < 0.05, two-tailed Student *t* test).

We first profiled the expression of 12 human ADAM isoforms that have been confirmed to possess enzymatic activities (Jones et al., 2016) in HCT116 cells and in APC-null (and more malignant) SW480 cells. As shown in Figure 1A, six ADAMs (ADAM8, ADAM20, ADAM21, ADAM28, ADAM30, and ADAM33) were not detectable in either type of cells, and three ADAMs (ADAM9, ADAM12, and ADAM15) were comparably expressed in both types of cells. ADAM10, ADAM17, and ADAM19 were significantly up-regulated in SW480 cells compared to HCT116 cells. Western blotting confirmed augmented protein expression of ADAM10/ADAM17/ADAM19. In addition, expression levels of ADAM10, ADAM17, and ADAM19 were elevated in human colorectal carcinoma biopsy specimens compared to adjacent non-tumorous tissues (Figure 1C). More important, there were elevated expression levels of ADAM10, ADAM17, and ADAM19 in tumor tissues as they became more malignant in terms of pathological grading (Figures 1D,E). It was also noted that elevated expression levels of BRG1, a chromatin remodeling protein, were detected in SW480 cells compared to HCT116 cells (Figures 1A,B), in the tumorous tissues compared to the adjacent tissues (Figure 1C), and in the highly malignant tumors compared to the lesser malignant tumors (Figure 1D), all consistent with the expression patterns of ADAMs. These data suggest that expression levels of certain ADAMs may correlate with CRC malignancies.

### ADAM Promotes CRC Cell Migration and Invasion

**FIGURE 2:**
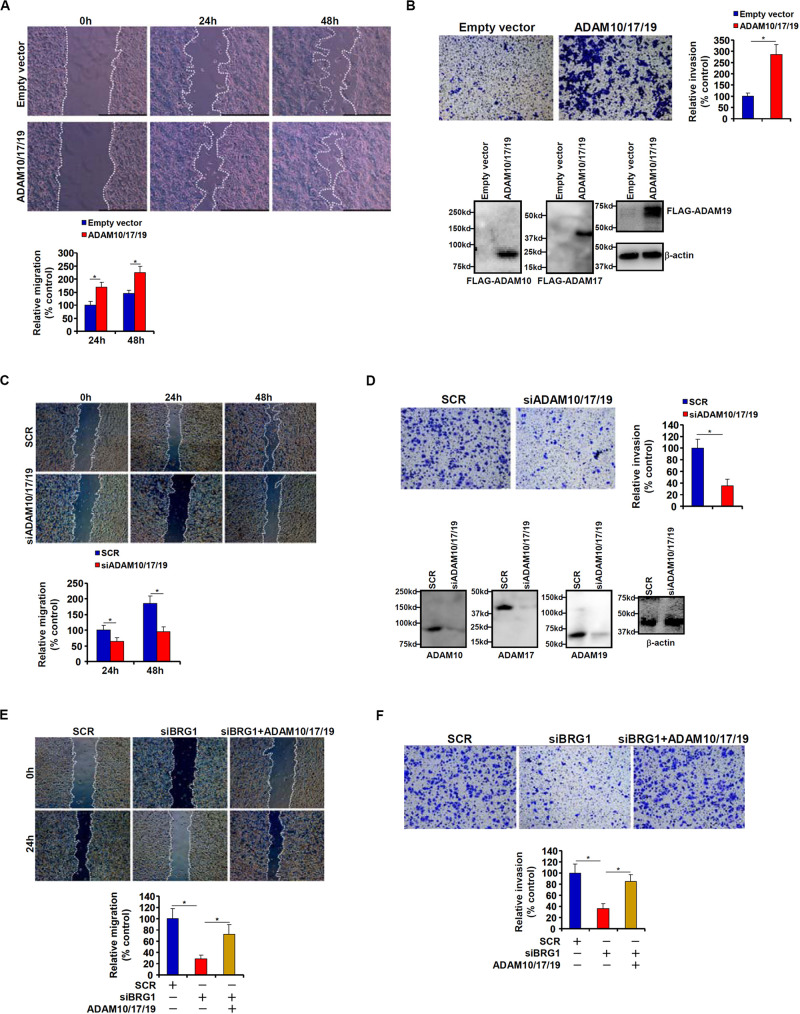
ADAM promotes colorectal cancer cell migration and invasion. **(A,B)** HCT116 cells were transfected with indicated expression constructs or an empty vector (EV). Cell migration was evaluated by wound healing assay **(A)**. Cell invasion was evaluated by Boyden Chamber assay **(B)**. Inset, Western blotting was performed with anti-FLAG and anti–β-actin. **(C,D)** SW480 cells were transfected with indicated siRNAs or scrambled siRNA (SCR). Cell migration was evaluated by wound healing assay **(C)**. Cell invasion was evaluated by Boyden Chamber assay **(D)**. **(E,F)** SW480 cells were transfected with indicated siRNAs in the presence or absence of ADAM expression constructs. Cell migration was evaluated by wound healing assay **(E)**. Cell invasion was evaluated by Boyden Chamber assay **(F)**. Data represent averages of three independent experiments, and error bars represent SEM (*p* < 0.05, two-tailed Student *t* test).

Because malignant CRC cells are characterized by the ability to metastasize owing to aggressive migration and invasion, we hypothesized that ADAM10/17/19 might be involved in the regulation of CRC cell migration and invasion. To test this hypothesis, expression constructs of ADAM10, ADAM17, and ADAM19 were transfected into HCT116 cells. Scratch-wound healing assay (Figure 2A) and *trans-*well assay (Figure 2B) showed that overexpression of ADAM10/17/19 considerably promoted the migration and invasion of HCT116 cells. On the contrary, depletion of ADAM10/17/19 in SW480 cells blocked migration (Figure 2C) and invasion (Figure 2D). Of interest, in the BRG1-high SW480 cells, knockdown of BRG1 by siRNA attenuated the migration (Figure 2E) and invasion (Figure 2F); reintroduction of ADAM10/17/19 into the cells restored migration and invasion. MTT assays suggested that neither ADAM overexpression nor ADAM knockdown influenced cell viability (Supplementary Figure S1). These data suggest that ADAM10/17/19, likely downstream of BRG1, may promote CRC cell migration and invasion.

### Wnt/β-Catenin Signaling Regulates ADAM Transcription

**FIGURE 3:**
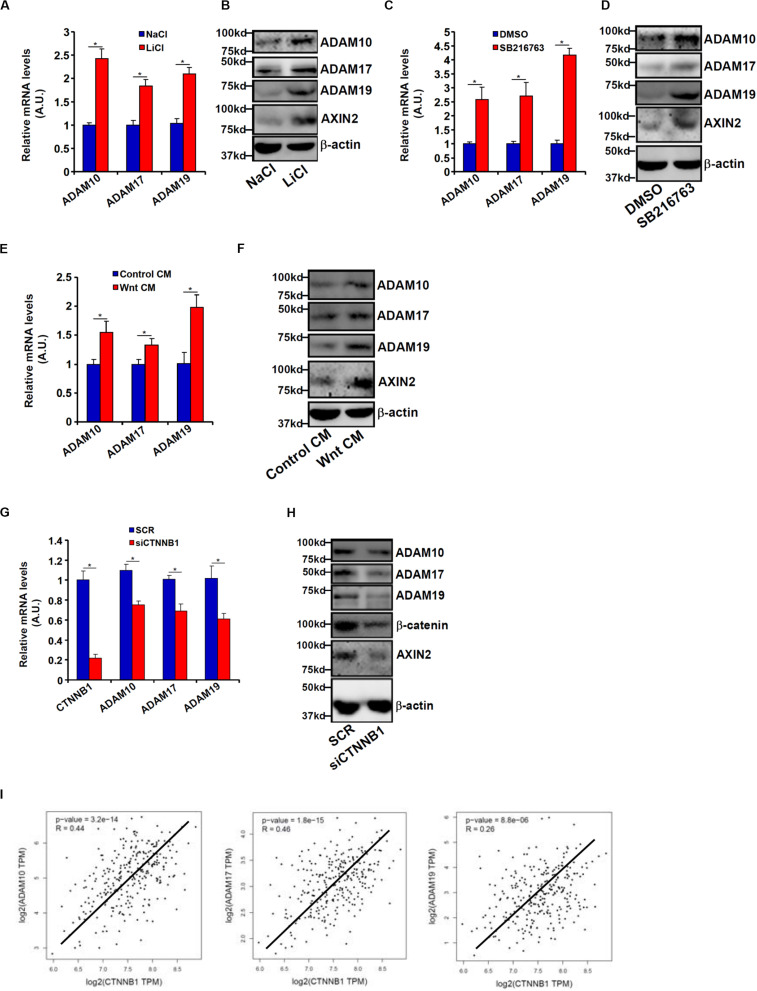
Wnt/β-catenin signaling regulates ADAM transcription. **(A,B)** HCT116 cells were treated with LiCl or NaCl for 8 h. ADAM expression levels were examined by qPCR and Western. **(C,D)** HCT116 cells were treated with SB216763 or dimethyl sulfoxide for 8 h. ADAM expression levels were examined by qPCR and Western. **(E,F)** HCT116 cells were treated with Wnt-containing CM or a control CM for 8 h. ADAM expression levels were examined by qPCR and Western. **(G,H)** SW480 cells were transfected with siRNA targeting β-catenin or scrambled siRNA (SCR). ADAM expression levels were examined by qPCR and Western. **(I)** Correlation between β-catenin expression and ADAM expression in CRC patients. Data represent averages of three independent experiments, and error bars represent SEM (*p* < 0.05, two-tailed Student *t* test).

Wnt signaling leads to stabilization and nuclear translocation of β-catenin, which serves as a key transcriptional regulator of genes involved in CRC metastasis. Previously, it has been shown that SW480 cells exhibit higher levels of β-catenin than HCT116 cells owing to a loss-of-function mutation in APC, a core component of the β-catenin destruction complex (Yang et al., 2006). We hypothesized that ADAM transcription might be activated by the Wnt signaling. HCT116 cells were treated with LiCl or SB216763, two different inhibitors for GSK3, which phosphorylates β-catenin and targets β-catenin for degradation; all three ADAMs were significantly up-regulated by treatment with LiCl (Figures 3A,B) or SB216763 (Figures 3C,D). In addition, exposure to the Wnt ligand-containing conditioned media (CM) also induced ADAM expression in HCT116 cells (Figures 3E,F). In contrast, depletion of β-catenin in SW480 repressed the expression of all three ADAMs (Figures 3G,H). Figure 3I showed that a strong positive correlation between β-catenin and ADAM expression levels was detectable based on the publicly disposed datasets^1^.

**FIGURE 4:**
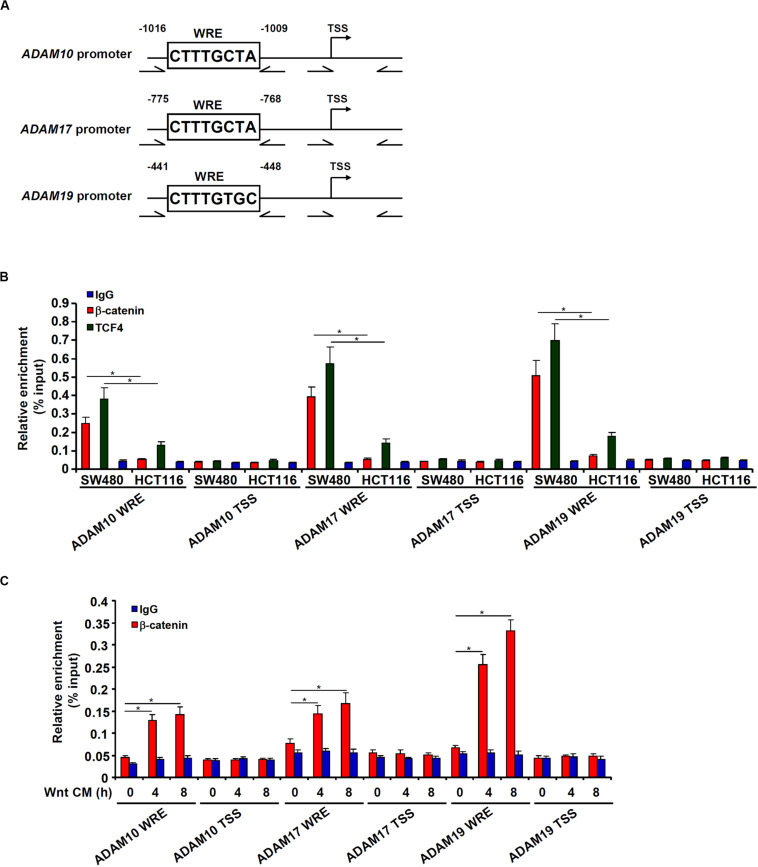
β-catenin directly binds to the ADAM promoters. **(A)** Schematic structures of the ADAM promoters with the WRE and the ChIP primers highlighted. **(B)** ChIP assays were performed in SW480 cells and HCT116 cells with anti–β-catenin, anti-TCF4, or IgG. **(C)** HCT116 cells were treated with Wnt-containing CM and harvested at indicated time points. ChIP assay was performed with anti–β-catenin or IgG. Data represent averages of three independent experiments, and error bars represent SEM (*p* < 0.05, two-tailed Student *t* test).

Bioinformatics analyses revealed a distinct Wnt response element (WRE) situated on each of three ADAM promoters (Figure 4A). Chromatin immunoprecipitation (ChIP) assays detected robust basal levels of β-catenin binding and TCF7L2/TCF4 binding on the ADAM promoters in SW480 cells compared to HCT116 cells (Figure 4B). However, stimulation of Wnt signaling with the CM strongly promoted recruitment of β-catenin to ADAM promoters in HCT116 cells (Figure 4C). Together, these data support a role for β-catenin in the transactivation of ADAM genes to promote CRC cell migration and invasion.

### BRG1 Regulates ADAM Transcription by Modulatingβ-Catenin Activity

**FIGURE 5:**
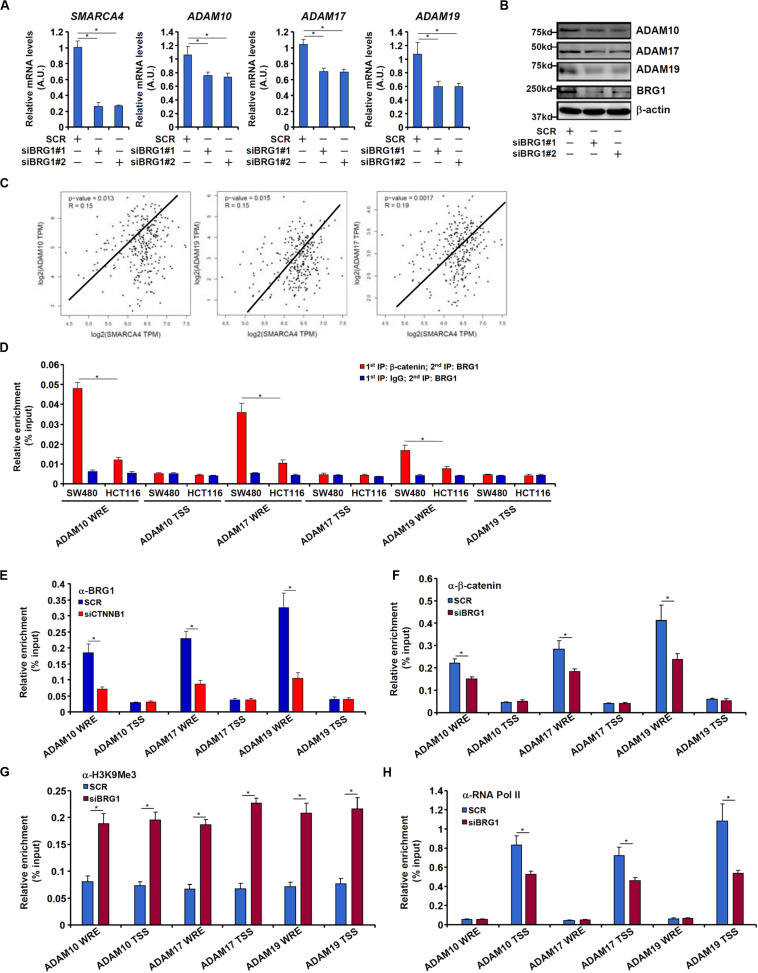
BRG1 regulates ADAM transcription by modulating β-catenin activity. **(A,B)** SW480 cells were transfected with siRNA targeting BRG1 or scrambled siRNA (SCR). ADAM expression was examined by qPCR and Western. **(C)** Correlation between BRG1 expression and ADAM10/17/19 expression in human colorectal cancer specimens. **(D)** Re-ChIP assay was performed with indicated antibodies in SW480 cells and HCT116 cells. **(E)** SW480 cells were transfected with siRNA targeting β-catenin or scrambled siRNA (SCR). ChIP assay was performed with anti-BRG1. **(F–H)** SW480 cells were transfected with siRNA targeting BRG1 or scrambled siRNA (SCR). ChIP assays were performed with anti–β-catenin **(F)**, anti-H3K9Me3 **(G)**, and anti-RNA polymerase II **(H)**. Data represent averages of three independent experiments, and error bars represent SEM (*p* < 0.05, two-tailed Student *t* test).

We have previously shown that the chromatin remodeling protein BRG1 activates Wnt signaling pathway to regulate liver regeneration by recruiting the histone demethylase KDM4 to modulate β-catenin activity (Li N. et al., 2019). We asked whether the same complex might be involved in ADAM transcription. Knocking down BRG1 expression with siRNAs in SW480 cells decreased mRNA (Figure 5A) and protein (Figure 5B) levels of ADAM10, ADAM17, and ADAM19. In tumor specimens collected from CRC patients, there was a positive correlation between BRG1 and ADAM10/17/19 (Figure 5C).

Re-ChIP assay revealed that a much stronger BRG1–β-catenin interaction could be detected across the WRE region, but not the transcription start site (TSS) region, of the ADAM promoters in SW480 cells than in HCT116 cells (Figure 5D). Depletion of β-catenin significantly suppressed BRG1 binding on the ADAM promoters (Figure 5E). Reciprocally, depletion of BRG1 dampened β-catenin binding to the ADAM promoters (Figure 5F). This might be attributed to increased levels of trimethyl H3K9, a repressive mark of transcription, detected not only surrounding the WRE region but also the TSS region of the ADAM promoters (Figure 5G). Of interest, occupancies of RNA polymerase II surrounding the TSS region were down-regulated by BRG1 deficiency (Figure 5H).

### BRG1 Recruits KDM4 to Activate ADAM Transcription

**FIGURE 6:**
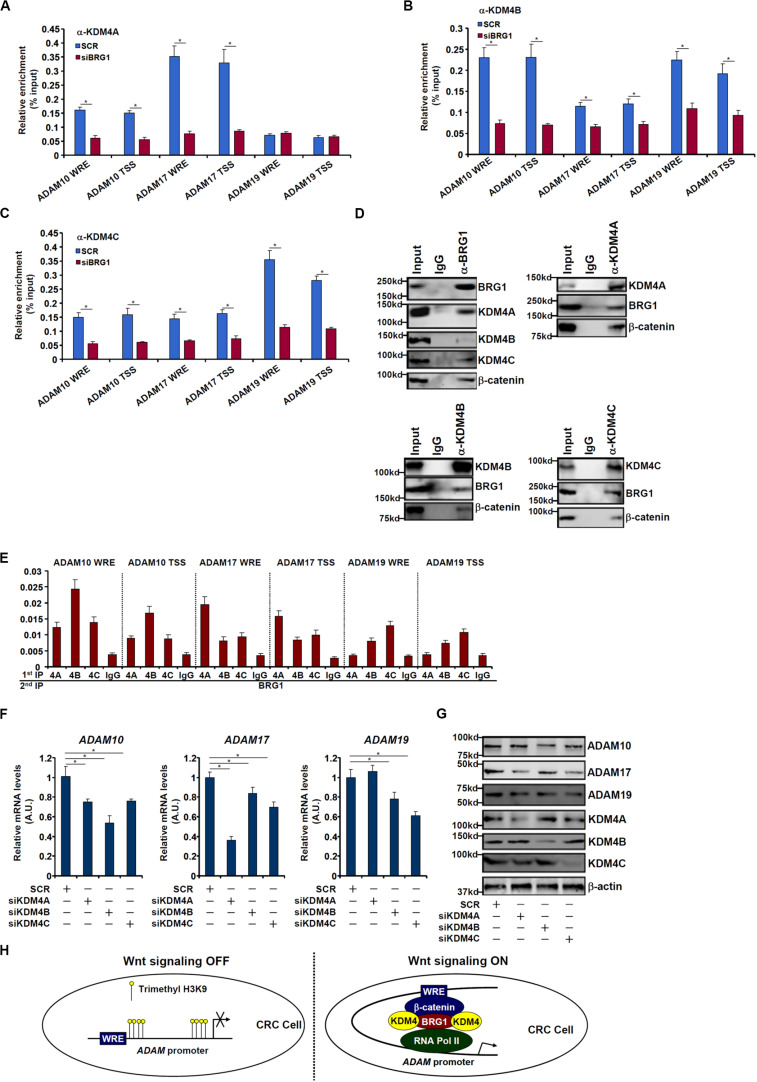
BRG1 recruits KDM4 to activate ADAM transcription. **(A–C)** SW480 cells were transfected with siRNA targeting BRG1 or scrambled siRNA (SCR). ChIP assay was performed with indicated antibodies. **(D)** Nuclear lysates were extracted from SW480 cells. Immunoprecipitation was performed with indicated antibodies. **(E)** Nuclear lysates were extracted from SW480 cells. Re-ChIP assay was performed with indicated antibodies. **(F,G)** SW480 cells were transfected with siRNA targeting KDM4 or scrambled siRNA (SCR). ADAM expression levels were examined by qPCR and Western. **(H)** A schematic model. Data represent averages of three independent experiments, and error bars represent SEM (*p* < 0.05, two-tailed Student *t* test).

We next performed ChIP assays to examine whether BRG1 may recruit KDM4 to the ADAM promoters. As shown in Figures 6A–C, there were great variances regarding the binding patterns of different KDM4 isoforms on the ADAM promoters. KDM4A binding was stronger on the ADAM17 promoter than the ADAM10 promoter but undetectable on the ADAM19 promoter (Figure 6A). KDM4B bound to the ADAM10 and ADAM19 promoters with comparable affinities, which were higher than the ADAM17 promoter (Figure 6B). KDM4C was detected to preferentially occupy the ADAM19 promoter (Figure 6C). BRG1 depletion largely abrogated the recruitments of KDM4 proteins to all the ADAM promoters. Immunoprecipitation assay using nuclear lysates extracted from SW480 cells confirmed that KDM4 proteins were in the same complex as BRG1 (Figure 6D). Furthermore, Re-ChIP assay showed that different KDM4–BRG1 complexes (Figure 6E) and KDM4–β-catenin complexes (Supplementary Figure S2) could be detected on the ADAM promoters. Consistent with the ChIP results, knockdown of different KDM4 isoforms exerted distinct effects on ADAM expression levels (Figures 6F,G). The changes in ADAM expression levels as a result of KDM4 knockdown mirrored the changes in trimethyl H3K9 levels on the ADAM promoters (Supplementary Figure S3).

## Discussion

CRCs have been one of the leading causes of cancer-related deaths for the last decade and remain a deadly pathology in the era of personalized medicine. Malignant forms of CRC are characterized by aggressive migration and invasion that enable cancer cells to spread to secondary sites. Here we describe a novel pathway in which the chromatin remodeling protein BRG1 promotes CRC cell migration and invasion by activating the transcription of ADAM proteinases (Figure 6H). We show that simultaneous overexpression of ADAM10/17/19 promoted, whereas knockdown of ADAM10/17/19 attenuated, CRC cell migration and invasion. These observations are consistent with previously published data that targeting ADAM10 (Atapattu et al., 2016), ADAM17 (Kyula et al., 2010), or ADAM19 (Zhang et al., 2015) with small-molecule compounds attenuates CRC development and progression in mice. Of note, ADAM10/17/19 overexpression compensated for BRG1 depletion to restore CRC cell migration and invasion indicating that BRG1 may be a common mediator of ADAM induction in malignant CRC cells. Indeed, ChIP assays confirmed that BRG1 could directly bind to the ADAM promoters by interacting with β-catenin. There have been a string of reports that consistently demonstrate a functional interplay between BRG1 and β-catenin. Griffin et al. (2011) are among the first to report that BRG1 is essential for the Wnt/β-catenin signaling by dictating the transcription of both Wnt receptor genes (*Frizzled*) and Wnt ligand genes (*Wnt*). A follow-up study by Weng and colleagues have found that the long non-coding RNA LncFZD6 recruits BRG1 to the *Frizzled6* promoter and the *Wnt5a* promoter to activate transcription, thereby driving liver cancer cell self-renewal (Chen et al., 2018). More recently, Li N. et al. (2019) and Wang et al. (2019) have independently reported that BRG1 deficiency cripples liver regenerative response in mice accompanying down-regulation of β-catenin target genes related to cell proliferation. In light of our new data as summarized here, it is tempting to propose that BRG1 may be an integral part of the Wnt/β-catenin pathway functioning at several different levels (upstream and downstream of β-catenin) to amplify the signaling cascade. This model can find support in a study published by Holik et al. (2014) in which BRG1 ablation in either mature epithelial cells (driven by the *Villin*-Cre) or in the intestinal stem cells (driven by the *Lgr5*-Cre) attenuates colorectal tumorigenesis and improves survival in *Apc*-deficient mice, which is attributable to the suppression of key β-catenin target genes involved in the initiation and progression of CRCs. As tantalizing as the model appears, it is not without a caveat. A recent investigation by Liu et al. (2019) has shown that BRG1 overexpression in epithelial cells (*Rosa*^Brg1/+^; *Villin*-Cre) protects, whereas BRG1 deletion in epithelial cells (*Brg1*^f/f^; *Villin*-Cre) exacerbates, colorectal carcinogenesis in a colitis-induced mouse model. The underlying mechanism, according to the authors, can be ascribed to the ability of BRG1 to regulate autophagy, which helps sequester reactive oxygen species and rein in aberrant inflammation, thus shielding the intestines from oncogenic attacks; it is not clear whether Wnt/β-catenin signaling pathway was altered by BRG1 gain of function or loss of function in this model. Therefore, BRG1 may act as both a promoter and suppressor of CRC pathogenesis, depending on the genetic background and the etiology, which advises against non-discriminate targeting of BRG1 in devising preventive and interventional approaches.

Our data suggest that BRG1 contributes to transcriptional activation of ADAM genes in part by recruiting H3K9 demethylases. In keeping with our observation, Berry et al. (2014), Chen et al. (2020), and Li N. et al. (2019) have recently reported that KDM4 can physically interact with β-catenin and potentiate the transcription of β-catenin target genes in CRC cells, glioblastoma cells, and hepatocytes, respectively. Of interest, BRG1 knockdown dampens the affinity of β-catenin for its target promoters pointing to a possible mechanism whereby BRG1 recruits KDM4 to erase H3K9 methylation and creates a friendlier chromatin environment for β-catenin to access L. This hypothesis can find support in several previously published studies. Li et al. (2017), for instance, have provided evidence to show that KDM3, a lysine demethylase with similar substrate preference as KDM4, stabilizes β-catenin binding on target promoters by altering the chromatin structure in CRC cells. Further, Suzuki et al. (2018), with the help of ChIP-seq techniques, have found that β-catenin selectively binds to target promoters abounded with high levels of acetylated H3K9, a histone marker antagonistic to methylated H3K9, in skeletal muscle cells. Whether BRG1 coordinates genome-wide histone modifications to modulate β-catenin binding in a functionally relevant manner awaits future investigations.

In summary, our data suggest that a β-catenin–BRG1–KDM4 transcriptional complex regulates ADAM10/17/19 expression. There are a few limitations regarding this study. First, although we have provided ample evidence to show that the β-catenin–BRG1–KDM4 transcriptional complex contributes to ADAM10/17/19 transcription, the underlying pathophysiological implication in CRC cell migration/invasion remains unclear. Additional experiments should be performed in additional cancer cells, authenticated in animal models of CRC metastasis, and verified in different, larger cohorts of patients. Second, the effects of KDM4 knockdown on ADAM expression in SW480 cells were rather modest pointing to alternative epigenetic mechanisms that might facilitate the regulation of ADAM transcription by β-catenin/BRG1. Third, the requirement of different KDM4s for global β-catenin–dependent transcription awaits further investigation. Clearly, further studies are needed before targeting this newly identified axis can be considered as a viable approach in personalized treatment of CRC patients.

## Materials and Methods

### Cell Culture, Plasmids, and Transient Transfection

Human CRC cells SW480 and HCT116 have been previously described (Sun et al., 2017). The Wnt3a-producing cells (CRL-2647^TM^) were purchased from ATCC. BRG1 expression constructs (Li et al., 2020a,b), FLAG-tagged ADAM10 expression construct (Parkin and Harris, 2009), ADAM17 expression construct (Granato et al., 2018), and ADAM19 expression construct (Chesneau et al., 2003) have been previously described. Transient transfections were performed with Lipofectamine 2000 (Invitrogen) as previously described (Mao et al., 2020;Yang et al., 2020).

### Human CRC Samples

All human studies were reviewed and approved by the Nanjing University of Chinese Medicine Committee on Ethical Conduct of Studies with Human Subjects. CRC tissues were collected, under informed consent, from surgical resection specimens of patients who had not undergone radiotherapy or chemotherapy in the Jiangsu Province Hospital of Chinese Medicine. Diagnoses of all cases were confirmed by histological examination. Tumor differentiation was graded by the Edmondson grading system.

### Protein Extraction, Immunoprecipitation, and Western Blot

Whole-cell lysates were obtained by resuspending cell pellets in RIPA buffer (50 mM Tris pH7.4, 150 mM NaCl, 1% Triton X-100) with freshly added protease inhibitor (Roche) as previously described (Shao et al., 2019; Weng et al., 2019). Specific antibodies or preimmune immunoglobulin G (IgG) (P.I.I.) was added to and incubated with cell lysates overnight before being absorbed by Protein A/G-plus Agarose beads (Santa Cruz). Precipitated immune complex was released by boiling with 1X sodium dodecyl sulfate (SDS) electrophoresis sample buffer. Western blot analyses were performed with anti-ADAM10 (Proteintech, 25900-1), anti-ADAM17 (Proteintech, 25209-1), anti-ADAM19 (Proteintech, 22216-1), anti-BRG1 (Abcam, ab110641), anti–β-catenin (Cell Signaling Tech, 8480), anti-KDM4A (Cell Signaling Tech, 5328), anti-KDM4B (Cell Signaling Tech, 8639), anti-KDM4C (Bethyl Laboratories, A300-885A), and anti–β-actin (Sigma, A2228) antibodies. All experiments were repeated three times.

### RNA Isolation and Real-Time Polymerase Chain Reaction

RNA was extracted with the RNeasy RNA isolation kit (Qiagen) as previously described (Zhao et al., 2019; Dong et al., 2020). Reverse transcriptase reactions were performed using a SuperScript First-Strand Synthesis System (Invitrogen). Real-time polymerase chain reactions were performed on an ABI Prism 7500 system with the following primers: *ADAM10*, 5′-GCTGTGATTGCCCAGAT ATCCA-3′ and 5′-CACCATGAAACTGATGTTACGGA-3′; *ADAM17*, 5′-CTTATGTTGGCTCTCCCAGA-3′ and 5′-CCAG GTCAGCTTCCTTTGTA-3′; *ADAM19*, 5′-AGAGTGTGGGT CCTGTGGTA-3′ and 5′-AACTGAACTGTTGCCTCAGC-3′. Ct values of target genes were normalized to the Ct values of a housekeeping control gene (18s, 5′-CGCG GTTCTATTTTGTTGGT-3′ and 5′-TCGTCTTCGAAACTCCG ACT-3′) using theDDCt method (Livak and Schmittgen, 2001) and expressed as relative mRNA expression levels compared to the control group, which is arbitrarily set as 1. All experiments were performed in triplicate wells and repeated three times.

### Scratch-Wound Healing/Migration Assay

Wound healing assay was performed as previously described (Yang et al., 2019b). Cells were resuspended in serum-free media. When the cells reached confluence, scratch wound was created by using a sterile micropipette tip. Cell migration was measured 24 h after the creation of the wound and calculated by Image Pro. Data were expressed as% migration compared to control arbitrarily set as 100%.

### Boyden Chamber Invasion Assay

Transwell assay was performed as previously described (Yang et al., 2019a). Twenty-four-well inserts (Costar) with 10 mg/mL Matrigel (Sigma) were used for invasion assays. Cells were resuspended in serum-free media and plated into the upper chamber with the lower chamber filled with complete media. Following exposure to indicated stimuli, the cells on the upper chamber were removed. Invaded cells were stained with 0.1% crystal violet and counted. Data were expressed as% invasion compared to control arbitrarily set as 100%.

### MTT Assay

Cell proliferation was measured using an MTT kit (Abcam) as previously described (Li Z. et al., 2019). Briefly, cells were plated in 12-well plates and allowed to attach overnight. Data were expressed as percentage proliferation compared to control arbitrarily set as 1.

### Chromatin Immunoprecipitation

ChIP assays were performed essentially as described before (Lu et al., 2019). In brief, chromatin in control and that in treated cells were cross-linked with 1% formaldehyde. Cells were incubated in lysis buffer (150 mM NaCl, 25 mM Tris pH 7.5, 1% Triton X-100, 0.1% SDS, 0.5% deoxycholate) supplemented with protease inhibitor tablet and phenylmethylsulfonyl fluoride. DNA was fragmented into ∼500-bp pieces using a Branson 250 sonicator. Aliquots of lysates containing 200 μg of protein were used for each immunoprecipitation reaction with anti–β-catenin (Cell Signaling Tech, 8480), anti-TCF4 (Cell Signaling Tech, 2569), anti-BRG1 (Abcam, ab110641), anti-trimethyl H3K9 (Millipore, 17-615), anti-RNA Polymerase II (Santa Cruz, sc-899), anti-KDM4A (Cell Signaling Tech, 5328), anti-KDM4B (Cell Signaling Tech, 8639), anti-KDM4C (Bethyl Laboratories, A300-885A), or preimmune IgG. For re-ChIP, immune complexes were eluted with the elution buffer (1% SDS, 100 mM NaCO_3_), diluted with the re-ChIP buffer (1% Triton X-100, 2 mM EDTA, 150 mM NaCl, 20 mM Tris pH 8.1), and subject to immunoprecipitation with a second antibody of interest. Precipitated genomic DNA was amplified by real-time PCR with the following primers:*ADAM10* WRE, 5′-AAAAGTTCTAGTTCATGAC-3′ and 5′-AAATTTATGTCACAGACTAAGGC-3′; *ADAM10* TSS, 5′-AAAGGAGGAAGGAAACGAAC-3′ and 5′-AAGCACCTCC CTCTCGCTCC-3′; *ADAM17* WRE, 5′-AAAGAGGCTCAGA AAAAGGAAC-3′ and 5′-AACACACCTTATTTTCC-3′; *ADA M17* TSS, 5′-AGCACTTAGTTCTTTGTGCC-3′ and 5′-AAAC TGGACCCCTCTCCGCAC-3′; *ADAM19* WRE, 5′-AAACCGG GAGATTGGAGTCC-3′ and 5′-ATTGACTACTCTTGGAGTC-3′; *ADAM19* TSS, 5′-AGAAGCCCTGAAGGGCTAG-3′ and 5′-AGGTTTTCAAGCAAAC-3′. A total of 10% of the starting material is also included as the input. Data are then normalized to the input and expressed as% recovery relative the input. All experiments were performed in triplicate wells and repeated three times.

### Statistical Analysis

Sample sizes reflected the minimal number needed for statistical significance based on power analysis and prior experience. Two-tailed Student *t* test was performed using an SPSS package. Unless otherwise specified, *p* values smaller than.05 were considered statistically significant.

## Data Availability Statement

The original contributions presented in the study are included in the article/Supplementary Material, further inquiries can be directed to the corresponding author.

## Ethics Statement

All human studies were reviewed and approved by the Nanjing University of Chinese Medicine Committee on Ethical Conduct of Studies with Human Subjects. Colorectal cancer tissues were collected, under informed consent, from surgical resection specimens of patients who had not undergone radiotherapy or chemotherapy in the Jiangsu Province Hospital of Chinese Medicine.

## Author Contributions

YF and LS conceived the project. LS, BC, JH Wu, CJ, ZW Fan, YF, and YX designed the experiments. LS, BC, JH Wu, CJ, and ZW Fan performed the experiments and collected and analyzed the data. YX wrote the manuscript. LS provided the funding. All authors contributed to the article and approved the submitted version.

## Conflict of Interest

The authors declare that the research was conducted in the absence of any commercial or financial relationships that could be construed as a potential conflict of interest.

## Footnotes

1 http://gepia.cancer-pku.cn
