# Supplementary material for: Epigenetic Regulation of a Disintegrin and Metalloproteinase (ADAM) Transcription in Colorectal Cancer Cells: Involvement of β-Catenin, BRG1, and KDM4
Source: Front Cell Dev Biol. 2020 Sep 11;8:581692. doi: 10.3389/fcell.2020.581692 (PMC7517301; doi:10.3389/fcell.2020.581692)
Supplement: Supplementary file 1 [file Image_1.pdf]

Sun L et al: ***Epigenetic regulation of a disintegrin and metalloproteinase (ADAM) transcription in colorectal cancer cells: involvement of β-catenin, BRG1, and KDM4***

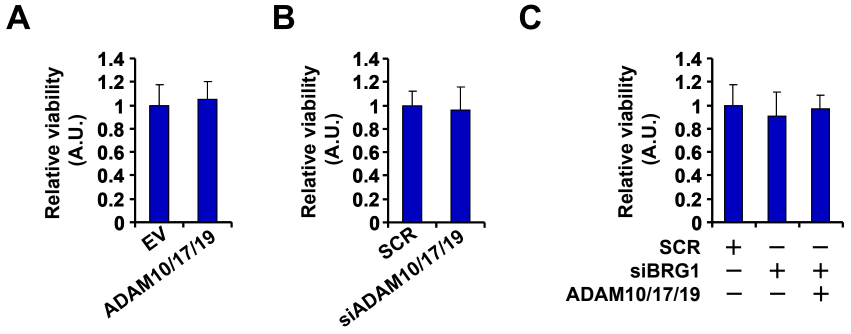

Fig.S1: (**A**) HCT116 cells were transfected with indicated expression constructs or an empty vector (EV). Cell viability was measured by MTT assay. (**B**) SW480 cells were transfected with indicated siRNAs or scrambled siRNA (SCR). (**C**) SW480 cells were transfected with indicated siRNAs in the presence or absence of ADAM expression constructs.

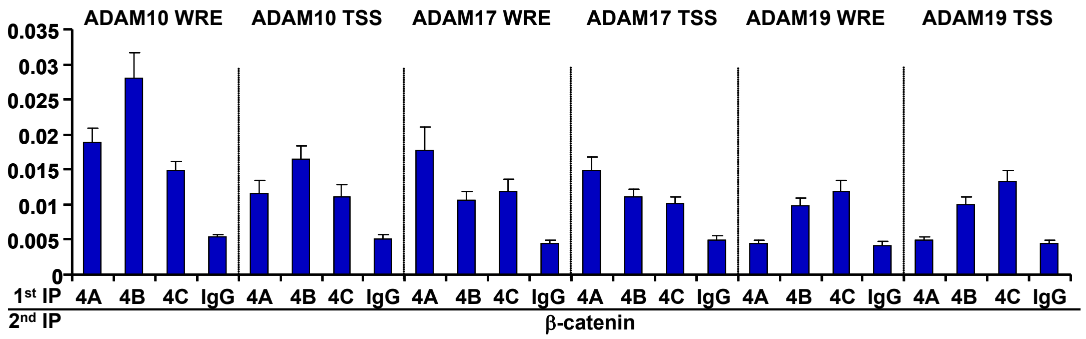

Fig.S2: Nuclear lysates were extracted from SW480 cells. Re-ChIP assay was performed with indicated antibodies.

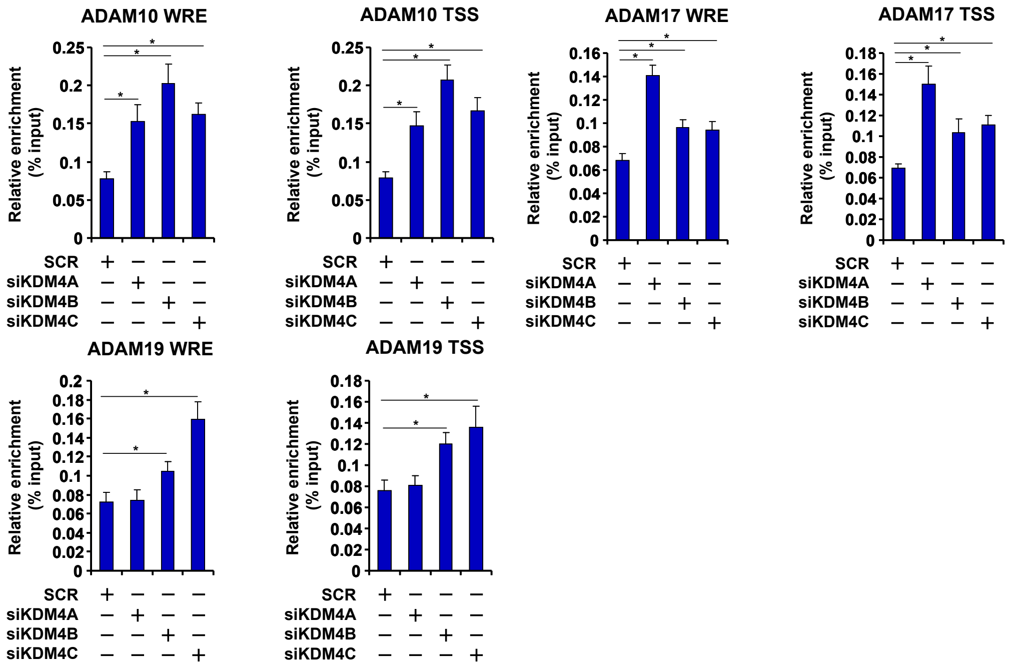

Fig.S3: SW480 cells were transfected with siRNA targeting KDM4 or scrambled siRNA (SCR). ChIP assays were performed with anti-H3K9Me3.
